# Intraoperative cholangiography still a current investigation

**Published:** 2013-12-25

**Authors:** C Iorga, S Cirimbei, V Strambu, F Popa

**Affiliations:** * ”Carol Davila” University of Medicine and Pharmacy, Bucharest, Department 10 – Surgery, “Dr. Carol Davila” Clinical Nephrology Hospital, General Surgery Clinic; ** I Oncological Surgery, “Prof. Dr. Alexandru Trestioreanu” Oncological Institute, Bucharest

**Keywords:** intraoperative cholangiography, iatrogenic bile duct injures

## Abstract

Abstract

Laparoscopic cholecystectomy is the standard treatment for patients requiring gallbladder removal.

Although the advantages of the laparoscopic technique are widely accepted, the introduction of this technique has doubled the rate of iatrogenic lesions of extrahepatic bile ducts.

Research methods for biliary tree also evolved, but intraoperative cholangiography, the traditional exploring method used for the biliary tree in classic cholecystectomy remains a valuable investigation in the laparoscopic technique.

We performed a retrospective study on a group of patients who underwent cholecystectomy (laparoscopic or classic). Of these, intraoperative cholangiography was performed on a total of 108 patients. Patients who underwent cholangiography motivated by preoperative investigations were excluded from the group and the study operates on patients in whom the decision to perform cholangiography was taken during surgery (45 cases).

We have analyzed the criteria that led to the motivation investigation (dilated cystic duct, suspected biliary duct stones, suspicion of iatrogenic biliary injury) results and subsequent therapeutic conduct.

The results showed that in 90% of the patients, the suspected diagnosis was confirmed by cholangiography (10 cases with normal cholangiography aspect, oddita 9 cases, 11 cases with bile duct stones, 2 cases with biliary tumor and 13 cases of iatrogenic biliary injury).

In conclusion, the decision to perform intraoperative cholangiography proved salutary, the suspected diagnosis was confirmed and the course of treatment was adjusted accordingly.

## Aim

The research methods (pre, intra and postoperative) for biliary tree evolved (ultrasound, intraoperative ultrasound, laparoscopic ultrasound, choledocoscopy, ERCP, cholangioMRI) but the intraoperative cholangiography - the traditional method used in the biliary tree exploration during classic cholecystectomy remains a valuable investigation even for laparoscopic cholecystectomy. 

## Introduction

 Intraoperative cholangiography was first introduced by Mirizzi in 1931, claiming the need to perform it routinely (1932) in order to detect biliary pathology. Although the first intention of performing routine intraoperative cholangiography was to diagnose bile duct stones, one of the advantages is the possibility of performing cholangiography for diagnosis or prevention of iatrogenic biliary lesions. 

 Discussions in literature over the last 20 years still remain open regarding the results of using selective / routine intraoperative cholangiography with a revival following the introduction of new technology: single-port cholecistectomy, NOTES. 

 Nowadays cholecystectomy = laparoscopic cholecystectomy in most cases. Laparoscopy is the standard treatment for patients requiring gallbladder removal. 

 NIH Consensus Conference in 1992 determined that “a patient with symptomatic gallstones is a candidate for laparoscopic cholecystectomy, if he is able to tolerate general anesthesia and has no serious cardiopulmonary diseases or other comorbid conditions that preclude the operation”. These indications include but are not limited to symptomatic gallstones, biliary dyskinesia, acute cholecystitis and complications related to this cholecystectomy stones including acute biliary pancreatitis. 

 Although the advantages of the laparoscopic technique are generally accepted, the introduction of this technique doubled the rate of iatrogenic lesions of the biliary duct. Although the number of cholecystectomies performed worldwide is growing, especially after the introduction of minimally invasive techniques (laparoscopic or endoscopic), the morbidity and mortality rates remains high. The rate of iatrogenic biliary lesions is 0.4-0.6% in laparoscopic cholecystectomy compared to the open path (0.2-0.3%). Some authors are even more drastic, saying that the rate of biliary duct lesions is three to five times higher in laparoscopic cholecystectomy than in the open technique. 

 In this context, there have been efforts to prevent, early diagnose and optimally treat these lesions. There is no widely accepted consensus for all to achieve all these goals. 

 In terms of prevention of these injuries the question that the world has to answer is not whether, but how it can be prevented? 

## Materials and methods 

 The retrospective study was performed on a sample of 2152 patients who underwent cholecystectomy (laparoscopic 23%, classical 77%), in the General Surgery Clinic of “Sf. Pantelimon” Emergency Hospital and the Surgical Clinic of “Carol Davila” General Nephrology Hospital, between the years 2002 and 2012.

 From the total number of cholecystectomies performed in this period and in these two clinics patients who received surgical intervention for acute abdomen, biliary pancreatitis and who underwent cholecystectomy during surgery for another pathology (cholecystectomy in patients with gastric cancer, colonic tumor invading gallbladder pancreaticoduodenectomy, etc.) were excluded. Only patients with preoperative diagnosis of symptomatic gallstones with indication for classical or laparoscopic cholecystectomy were selected.

 Of this total number, intraoperative cholangiography was performed for 108 patients (5%). Patients who underwent cholangiography motivated by preoperative investigations (jaundice, acute pancreatitis or angiocholitis, history of known or suspected bile duct stones) were excluded from the group; the study unfolding on the cases in which the decision was made intraoperatively to perform cholangiography (45 patients, representing 2.09%).

 Of these 45 patients, 33 were operated by open technique, in 12, the operation began laparoscopically but in 7 cases it was converted before performing cholangiography. It should be noted that although in the first years of the period studied almost all the patients were operated by open technique, the proportion of laparoscopic surgery has increased since 2007, so that in the last two years, most interventions have been laparoscopic. Also, in the event of the introduction of laparoscopic technique, a high proportion of conversions were motivated by the need to perform intraoperative cholangiography. The 5 cases who underwent laparoscopic intraoperative cholangiography took place in the period 2011-2012.

 The results obtained by cholangiography were: 10 cases with normal cholangiography aspect, 9 cases of odditis, 11 cases with bile duct stones, 2 cases with biliary tumor and 13 cases with iatrogenic biliary injury.

 One possible conclusion is that performing intraoperatively motivated cholangiography has a diagnostic value for biliary lesions in 13 cases and the appearance of the value of prevention in 10 cases is normal.

 40 patients were operated by open technique (including the converted cases), the results were the following: 9 cases with odditis, 9 cases with bile duct stones, 2 cases of tumor, 12 cases with lesion of the biliary duct and 8 normal results.

 In 5 patients who were operated laparoscopically we discovered two cases of bile duct stones, 2 results were normal and 1 with iatrogenic injury of bile ducts.

 For the cases in which the investigation had normal results (8 +2), surgery went on without technical modifications. The criteria that motivated the investigation were the following: the presence of a dilated cystic duct, difficulty in recognizing the local anatomy because of the reshuffling caused by local inflammation and the diffuse bleeding in the operating field.

 The 13 cases of iatrogenic biliary lesions were divided according to Strasberg classification:

 - Type A - 5 cases (3 slip ligation, 2 cases of aberrant duct bile leak)

 - TYPE D - 5 cases

 - TYPE E - 3 cases, 1 case for each subtype E1, E3 and E4

 In cases of iatrogenic lesions of biliary tract, cholangiography was motivated by the presence of bile leak in the operating field and the “change” in the local anatomy (main biliary duct clipped and cut followed by the retraction of the distal end, identifying a second “cystic”, actually the right hepatic duct which was clipped).

 In these cases, intraoperative cholangiography had a diagnostic value for iatrogenic biliary lesions.

 Summarizing, the intraoperative cholangiography revealed 13 iatrogenic lesions of the biliary duct which were already produced (0.6% of all cholecystectomies performed) and prevented the potential incidence of injury in 0.46% of cases.

## Discussion

 Intraoperative cholangiography has a definite diagnostic value in terms of biliary pathology.

 The value to prevent the iatrogenic biliary injury is brought into question.

 There are numerous studies for or against performing routine intraoperative cholangiography or on selectable criteria.

 The performance of routine intraoperative cholangiography has three major advantages: the identification of anatomical structures of the biliary tree and therefore the prevention of their lesion, identifying duct stones unsuspected preoperatively and concomitant training of the surgical team in performing the maneuver. However, the correct identification of the anatomy although it decreases the rate of extrahepatic biliary lesions is not a guarantee for preserving the integrity of these structures. Even in the case of experienced surgeons and supporting cholangiography routinely, the rate of iatrogenic biliary injury is not null.

**Fig. 1:**
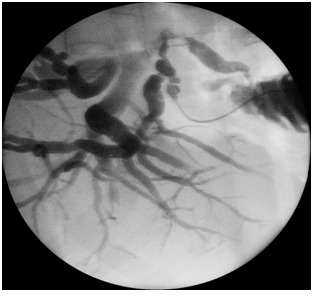
Intraoperative cholangiography – common bile duct stenosis, cystic duct implanted in the right hepatic duct

 Proponents argue about the need to perform routine cholangiography because it leads to the identification of anatomical abnormalities and the avoidance of biliary injury. Several observational studies demonstrate that the rate of iatrogenic injury can be reduced by 50% (Australia in 1988-1994 [**1**]), or on the contrary, a study of 30 630 discharged patients who underwent cholecystectomy during 1991-1998, Washington, showed a rate of biliary lesions 61% higher, in which cholangiography was not performed [**2**]. Also, it may limit an injury that already occurred (e.g. avoid the sectioning of the main biliary duct after clipping it in the wrong way).

 The continuous performance of routine cholangiography results in the lengthening of the operative time (mean time of 30 minutes) and the increase in the costs of the intervention. It can also have false positive results in 1-3% of cases, resulting in non-needed exploration of biliary tract.

 The training of the surgical team in performing the cholangiography can be an advantage especially during the learning curve, when it is important to correctly view the local anatomy, but, in the hands of an experienced surgeon, an accurate dissection especially where the cystic duct flows into CBP, can replace this investigation. The estimated time needed to perform the cholangiography versus the dissection of cystic duct is comparable.

 Considering all these arguments, the use of routine cholangiography is not justified.

 An Italian study [**3**] conducted on 56 591 cholecystectomies performed during 1998-2000 showed an incidence of approximately 0.42% of iatrogenic lesions without finding statistically significant differences between cases in which cholangiography was performed routinely or selectively (though the rate of injuries was 0.32% in cases of routine cholangiography compared to 0.43% in cases in which it was done selectively). Nearly a quarter of cases diagnosed with iatrogenic lesions were found in these centers before performing the cholangiography, so the rate lesion is much smaller.

 An average of 750 000 cholecystectomies are done in U.S. per year; only 27% of the surgeons routinely perform intraoperative cholangiography, the rest using it only in cases with suspected bile stones or iatrogenic injury, or believing that the patient has an increased risk for the production of an injury [**4**].

 In the selected cases, carrying selectable cholangiography can bring some advantages. There is not an established consensus showing clear indications for the performance of selective cholangiography. Certain statements found intraoperatively: unclear anatomy, stones in the cystic duct, cystic dilated for more than 4 mm, are widely accepted indications. The performance of intraoperative cholangiography can reduce the rate and severity of iatrogenic biliary lesions and may help to an earlier diagnosis (SAGES Guidelines).

## Conclusion

We support the need to perform intraoperative cholangiography on a selective basis (known and widely accepted criteria and possibly introduce new ones) because we believe that if you perform this maneuver it could result in the avoidance of such cases (in the experience of each surgeon in part) of lesion of biliary duct (with known consequences) then, this investigation is not pointless.
